# Assessing Emotion and Sensitivity of AI Artwork

**DOI:** 10.3389/fpsyg.2022.879088

**Published:** 2022-04-05

**Authors:** Ujué Agudo, Miren Arrese, Karlos G. Liberal, Helena Matute

**Affiliations:** ^1^Departamento de Psicología, Universidad de Deusto, Bilbao, Spain; ^2^Laboratorio de intervención, Bikolabs/Biko, Pamplona, Spain

**Keywords:** human–computer interaction, bias, stereotype, music, art, artificial intelligence

## Abstract

Artificial Intelligence (AI) is currently present in areas that were, until recently, reserved for humans, such as, for instance, art. However, to the best of our knowledge, there is not much empirical evidence on how people perceive the skills of AI in these domains. In Experiment 1, participants were exposed to AI-generated audiovisual artwork and were asked to evaluate it. We told half of the participants that the artist was a human and we confessed to the other half that it was an AI. Although all of them were exposed to the same artwork, the results showed that people attributed lower sensitivity, lower ability to evoke their emotions, and lower quality to the artwork when they thought the artist was AI as compared to when they believed the artist was human. Experiment 2 reproduced these results and extended them to a slightly different setting, a different piece of (exclusively auditory) artwork, and added some additional measures. The results show that the evaluation of art seems to be modulated, at least in part, by prior stereotypes and biases about the creative skills of AI. The data and materials for these experiments are freely available at the Open Science Framework: https://osf.io/3r7xg/. Experiment 2 was preregistered at AsPredicted: https://aspredicted.org/fh2u2.pdf.

## Introduction

In recent years Artificial Intelligence (AI) has started to contribute to areas and domains that until now were associated solely with human abilities (Wegner and Gray, 2017), such as writing novels (Jozuka, 2016), painting pictures (Christie’s, 2018), devising magic tricks (Williams and McOwan, 2014), or composing music (Adams, 2010; Deah, 2018). However, there is not much empirical evidence on how people perceive the skills of AI in these domains, particularly in the field of art, which is the focus of the present research.

For example, in the case of music, critics and audiences seem not have received the contribution of AI very well. Let us take the case of David Cope as an example. He is a Professor at the University of California who has been generating musical compositions through Artificial Intelligence for more than two decades. His first shows to the public, a piece of music similar to those of Bach, at a contest at the University of Oregon, and another piece with the style of Mozart at the Santa Cruz Baroque Festival (Johnson, 1997), were received with rejection, contempt and even wrath (Friedel, 2018). Cope was unable to have recognized musicians play his compositions publicly, not even years later (Saenz, 2009). The critics described his work as mere imitation, lacking in meaning and soul (Johnson, 1997). Since then, the current technological advancements do not seem to have changed the perception of the artistic ability of AI, at least in the context of classical music. The dissatisfied reactions of the public and the negative criticisms received by the conclusion of the unfinished symphonies of Mahler (Zappei, 2019) and Schubert (Mantilla, 2019) by AI, confirm this general rejection.

This rejection of the artwork of the machine has also been found in the laboratory as indicated by the few existing studies on the subject. For instance, Ragot et al. (2020) asked a large sample of participants to evaluate a series of paintings created by humans or by AI. Those created by humans were evaluated more positively than those created by AI in terms of linking, beauty, novelty, and meaning.

Several studies that use a modified form of the Turing Test as their procedure, also report undervaluation of AI artwork. For example, Moffat and Kelly (2006) showed some musical pieces to a small sample of participants. They asked their participants to evaluate the musical pieces and try to guess if they had been composed by humans or by computers. Regardless of the genre that they listened to, the participants preferred the works that they guessed that had been composed by humans. Similarly, Chamberlain et al. (2018) presented several works of visual art created by humans or by computers and asked their participants to guess their authorship and to evaluate them. When participants liked the artworks, they assumed that the artist was a human. These studies suggest that perhaps the general preference for the artwork created by humans might not rest on the objective quality of the artwork but on the prejudices that people may have against music created by machines.

Importantly, in the aforementioned studies, it is not possible to conclude whether the undervaluation of artworks is due to the artwork itself or to prejudices against the capacity of AI in its role as an artist. According to Sundar (2020), people associate certain negative traits and stereotypes with machines, such as more inflexibility, emotionlessness, and coldness. For example, in a study concerned with how people vale the authenticity of artwork created by AI, Jago (2019) observed that the evaluations of authenticity of the artwork created by AI were better when the experimental participants believed that a human had created the artwork. The author used two different measures of authenticity: (a) type authenticity (i.e., whether the artwork was considered authentic so that it could be classified as art); and (b) moral authenticity (i.e., whether it reflected the values or motivations of its creator). According to this research, when participants believed that the artist was a human, they rated it as more authentic than when they knew that it was the artwork of an AI algorithm, but only in terms of moral authenticity, not type authenticity. That is, participants accepted that the AI algorithm’s work was authentic and could be classified as art, but did not consider that the artwork was authentic in the sense of reflecting the artist’s values, motivation, or essence.

In this line, in a recent study, Hong et al. (2020) measured the perceived quality of musical pieces composed by AI. Once it was known that the piece of music to be heard had been composed by an AI or by a human, the participants listened to the piece. Then they rated its quality in terms of aesthetic appeal, creativity and craftsmanship. The participants also indicated what their attitudes toward creative AI were, and to what extent their pre-listening expectations had been violated. Even though the design of this study included both AI music and human music, the authors did not address whether the artwork of AI and that of humans was valued differently. Nevertheless, they concluded that acceptance of the creative skills of AI would be a necessary requirement for a positive evaluation of their artistic performance.

The discomfort produced by the inclusion of machines in the artistic context could be related to a more general phenomenon known as “algorithm aversion,” observed in decision-making (Shaffer et al., 2013; Dietvorst et al., 2015; Castelo et al., 2019; Yeomans et al., 2019). According to the literature on aversion, people would distrust the recommendation of AI algorithms even in cases when their advice is better than that of humans. However, this is not a simple phenomenon. For example, Agudo and Matute (2021) observed that algorithmic explicit recommendations were able to influence voting preferences, but not dating preferences. Quite possible people may see political decisions as something rational, and therefore susceptible of improvement through algorithm recommendation. Dating preferences, by contrast, may be regarded as something more subjective and free of rationality, which might explain the participants’ resistance to explicit recommendation from machines on this domain. Indeed, the algorithms used by Agudo and Matute were actually able to influence dating preferences, but only when the recommendations were not explicit, but covert (e.g., increasing the number of presentations of certain candidates over the other ones in order to make them look more familiar).

As a counterpoint, the work of other composing AI artists has received better criticisms. This is the case of the death metal Dadabots band, composed by an artificial neural network, that sums a total of 10 albums in the market (Merino, 2019). That is, in some areas, the performance of machines seems to be valued better than that of humans. For example, Liu and Wei (2019) found that news articles written by an algorithm were considered more objective and less emotionally involved than those written by humans. This could be due to the fact that machines are associated to, in addition to negative stereotypes, also to positive traits, such as objectivity, lack of bias, and neutrality (Sundar, 2008). These stereotypes would mean an overvaluation of machine performance over that of humans in some areas, a phenomenon known as *algorithmic appreciation* (Logg et al., 2018), which would cause the opposite effect to the aforementioned phenomenon of aversion to the algorithm.

Despite these contradictory views on the human response to the performance of AI in art, and despite their wide penetration in art, as Chamberlain et al. (2018) state, there is little understanding of how society reacts to AI in the arts and there is not enough research that addresses in psychological terms the relationship between human–computer interaction (HCI) and aesthetics.

For these reasons, the purpose of our research was to test, first, whether people actually report a different experience of art when they know it was created by AI as compared to when they believe it was created by a human. And second, whether that differential assessment could be attributed to a differential quality of the artwork, or could be due just to prejudices or biases about the authorship. To this end, our Experiment 1 was designed to test whether people exposed to an identical piece of art, composed by AI, attributed a different sensitivity and emotion if they knew the artist was AI, as compared to when they believed the artist was human. The purpose of Experiment 2 was twofold. First, it was designed to replicate Experiment 1. Even though it will reproduce the basic features of Experiment 1, we consider good practice to obtain convergent results in more than one experiment. In addition, Experiment 2 will also extend the results of Experiment 1 by testing a slightly different setting, a different artwork, and by adding some additional measures.

Unlike previous studies in which participants compared human artworks vs. artworks performed by AIs, in both experiments all of our participants were exposed only to artwork created by an AI. The critical manipulation was that some of them were told that the artist was a human while some of them were told that it was an AI. We predicted that people would attribute AI a poorer ability than human artists to perform with sensitivity a piece of artwork and a weaker ability to evoke emotions in the audience. To the best of our knowledge, this hypothesis has not yet been tested.

## Ethics Statement

The Ethics Review Board of the University of Deusto approved the procedure for these experiments as part of a larger project on The Influence of Algorithms on Human Decisions and Judgements. Written informed consent was not requested because the research was online and harmless, participation was anonymous, and participants submitted their responses to the questionnaires voluntarily. No personal information was collected.

## Experiment 1

### Method

#### Participants and Materials

We recruited a sample of 249 participants (55% women, 8% unknown), through the snowball procedure using a WhatsApp message submitted to several groups in Spain. These groups also contributed to the spread of the message. The WhatsApp message was an invitation to participate in a study on “music and feelings.” It included a link to an online study that was conducted in Spanish and using the Qualtrics platform.

The computer program randomly assigned each participant to one of two groups: AI artist (*n* = 115), and human artist (*n* = 134). All participants watched the same video^1^ in which an AI improvised piano melodies while painting on canvas following the rhythm of the music, in which the author of the work is not seen (neither AI nor humans).

#### Design and Procedure

Table 1 shows a summary of the experimental design. After accepting the online informed consent, the participants read different instructions for each group before watching the video. These instructions were our experimental manipulation. We told group AI artist that the artist was an AI (*“We introduce you to WCMM, an Artificial Intelligence that improvises at the piano while painting on canvas” /in Spanish:"Te presentamos a WCMM, una Inteligencia Artificial que improvisa al piano mientras pinta sobre un lienzo”*), and we told group human artist that the artists were humans.

**Table 1:** Design summary of experiment 1.

Group	Instructions	Treatment	Questions
AI Artist	Artist is AI	Video	Emotion & sensitivity
Human artist	Artists are humans		

To create the artwork needed for this research we used a type of recurrent neural network, known as LSTM (Long Short Term Memory), which is a specialist in learning from sequences and allows us to generate a polyphonic music improvisation with expressive tempos and dynamics. We did not specify to the participants the model or the type of AI that we used to create the artwork. We simply referred to it with the term artificial intelligent (AI), rather than other terms, such as neural network or algorithm, because AI is the term most similar to those used in the aforementioned literature in experiments with human participants.

On the other hand, the true authorship of the work was hidden from the other group and attributed to human artists. To control the genre of human artists, half of the participants in the human artist group were told that the composer and the painter were men (*“We introduce you to Javier Aldaz and Miguel Beltrán, two artists improvising on the piano while painting on canvas”/“Te presentamos a Javier Aldaz y Miguel Beltrán, dos artistas que improvisan al piano mientras pintan sobre el lienzo”*); and the other half was told that the composer and the painter were women (*“We introduce you to Ana Aldaz and María Beltrán, two artists improvising on the piano while painting on canvas”/“Te presentamos a Ana Aldaz y María Beltrán, dos artistas que improvisan al piano mientras pintan sobre lienzo”*).

We decided to use an audiovisual format instead of just audio format, as the artwork to be evaluated, combining musical and visual composition, to show the overwhelming creative capacity of AI today. Although the use of an audiovisual work implied that participants would evaluate the artwork at a multisensory level and this could have different effects than an exclusively auditory or visual piece, in this experiment we considered that a video where an AI improvises at the piano while painting on canvas would adequately show the current potential of AI and would better respond to the expectations of participants in the AI artist group, as they are likely to associate AI performance in art with multimedia works. In any case, an exclusively auditory piece was then tested in Experiment 2.

After watching the video, all participants were asked about the artists’ performance. Previous experiments have focused on the assessment of the AI-generated artwork (Hong et al., 2020) or on the audience experience (i.e., whether they liked the performance, e.g., Moffat and Kelly, 2006). Therefore, we decided to find out if there were any differences in the emotion evoked during the performance and the artists’ sensitivity when a non-expert audience thought the work had been performed by an AI or by humans. To this end, we used two simple questions. One question asked them to rate the emotion that the play had evoked in them (*“Now that you have seen the video of this Artificial Intelligence/ of these artists /of these artists, to what degree would you say that it arose your emotion?”*/*“Ahora que has visto el vídeo de esta Inteligencia Artificial/ de estas artistas/de estos artistas, ¿hasta qué punto dirías que te ha emocionado?*”). The other question asked them to rate the sensitivity that they attributed to the artist (*“And how would you rate the artist’s sensitivity?”*/“*¿Y cómo calificarías su sensibilidad?”*). As we were interested in the subjective assessment of a non-expert audience, we did not specify how participants should or should not understand the terms “emotion” and “sensitivity.” Instead, we simply let them provide their default, subjective, answers. The participants provided their answers to each of these two questions using a scale labeled from 0 to 10. The two ratings that they provided were our dependent variables.

We are aware that Internet-based research can in principle raise some concerns regarding important aspects, such as the quality of the audio received by each participant, or even the conditions in which many of them might have performed the experiment (e.g., at home vs. at a cafeteria). It should be noted, however, that our experimental design guarantees that any effect of random or unknown factors should affect both groups of participants equally. That is, because participants were randomly assigned to each group, the only difference between the groups was the independent variable, that is, that they received different instructions. Therefore, any differential results observed between the two groups in the sensitivity ratings or the emotional ratings should allow us to conclude that they are due to the differential instructions. We predicted that the ratings should be lower when participants were instructed that the artist was an AI.

### Results and Discussion

First, we made sure that there were no differences between cases in which the artists were women or men, neither with respect to the induced emotion [*M_Men_* = 4.19, *SD_Men_* = 2.66, *M_Women_* = 3.99, *SD_Women_* = 2.81, *t*(132) = 0.44, *p* = 0.659, *d* = 0.08] nor with respect to the attributed sensitivity [*M_Men_* = 5.84, *SD_Men_* = 2.44, *M_Women_* = 5.84, *SD_Women_* = 2.79, *t*(132) = 0.00, *p* = 1.00, *d* = 0.00]. Therefore, in the following analyses we collapsed the data of men and women artists in the human artist group.

**Figure 1:**
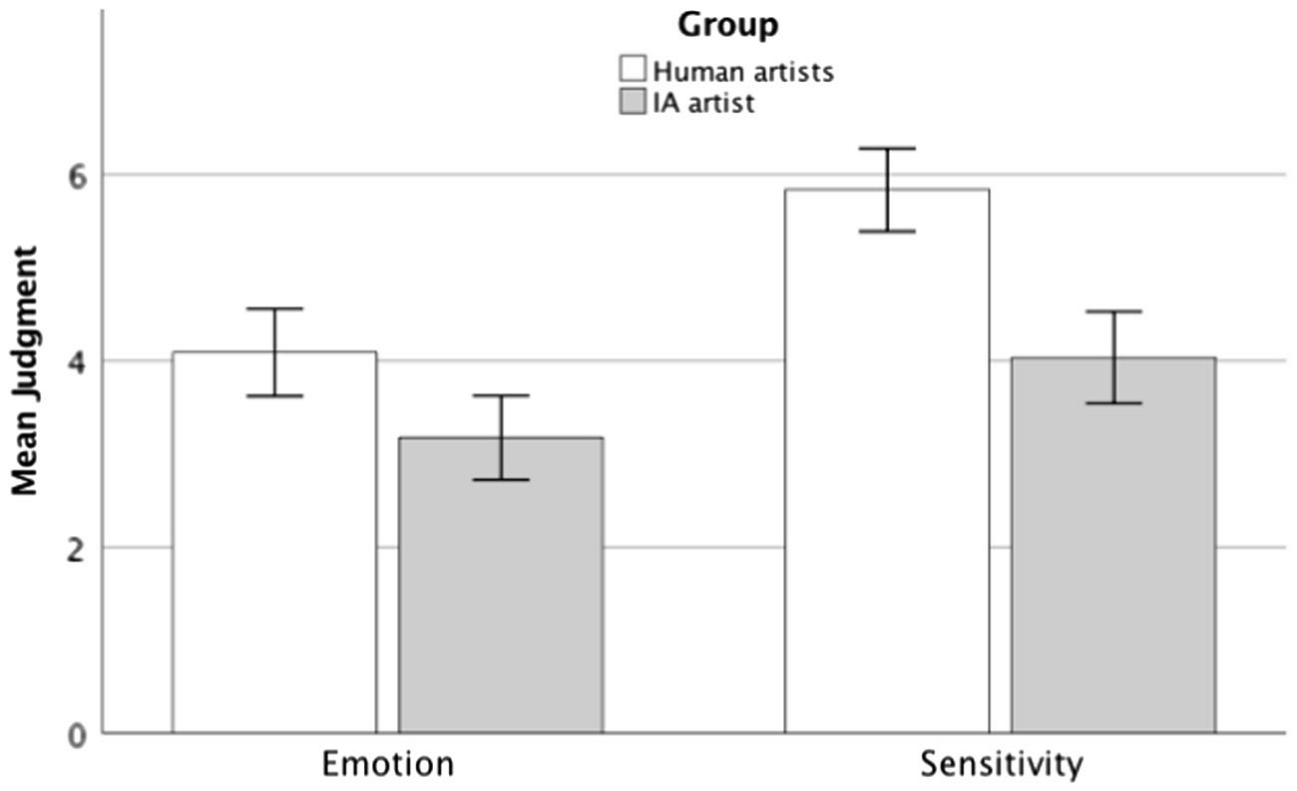
Judgment of artists’ evoked emotion and sensitivity by group in experiment 1. Error bars represent 95% CI.

Consistent with our hypothesis, and as can be seen in Figure 1, the participants reported stronger emotional arousal when they thought the artist was a human as compared to when they thought it was an AI. This was confirmed by a *T-Student*^2^ test for independent samples, *M_Human Artists_* = 4.09, *SD_Human Artists_* = 2.73; *M_AI artist_* = 3.17, *SD_AI artist_* = 2.45; *t*(247) = 2.76, *p* = 0.003, *d* = 0.35.

Likewise, this figure also shows that participants attributed stronger sensitivity to the artist when they thought it was human as compared to when they thought it was an AI, *M_Human Artists_* = 5.84, *SD_Human Artists_* = 2.61; *M_AI artist_* = 4.03, *SD_AI artist_* = 2.66; *t*(247) = 5.38, *p* < 0.001, *d* = 0.68, which is also consistent with our predictions.

The results of this experiment show that knowing that artificial intelligence has been the author of an audiovisual artwork seems to reduce the way in which the artwork is experienced and the artist is valued. These results replicate and extend previous findings on the different appreciation of the art created by humans or by AIs. Importantly, given that the artwork in our experiment was exactly the same in both groups, the results show that the participants’ assessments may not rest on the artwork itself but on their previous prejudices about the artists’ abilities.

## Experiment 2

This experiment aims to replicate the results of the Experiment 1, as well as to collect some additional variables that had been included in previous studies, in order to further facilitate the comparison of results and the implications of the present research.

Not many studies have been conducted on how people judge the art generated by artificial intelligence. Most of those studies focus on testing whether the machine can exhibit a behavior as a composer that is indistinguishable from that of a human, with a similar test to the Turing test but in the context of art (Yang et al., 2017). Other studies focus on evaluating whether there are differences in the quality of the musical compositions created by computer models (Pearce and Wiggins, 2007; Chu et al., 2017). The few studies that do focus on evaluating people’s experience with AI-created art, such as our Experiment 1, differ considerably in terms of purposes and methods used.

While some studies reveal the authorship of the AI before the participants judge the artwork (Hong and Curran, 2019; Hong et al., 2020; Ragot et al., 2020), as is the case in our experiments, other studies report it after collecting the participants judgments, therefore using a procedure similar to the aforementioned Turing test (Moffat and Kelly, 2006; Chamberlain et al., 2018).

Moreover, there is no uniformity in the variables collected in these studies. Some studies use judgments on the quality of the artwork as their main dependent variable. They may use measures defined by the authors themselves (e.g., originality or aesthetic value in Hong and Curran, 2019; and perceived beauty and meaning in Ragot et al., 2020). In the case of Hong et al. (2020), they used a validated 9-item scale, based on the original 18-item scale from Hickey (1999). This scale makes it easier for music teachers to evaluate their students’ compositions. Thus, its items require a certain professional knowledge of the subject and are very different from the items used by other researchers for the same variable. Furthermore, other studies focus on evaluating the experience of the participants rather than the quality of the artwork itself, with more subjective measures, such as attractiveness (Chamberlain et al., 2018), liking (Moffat and Kelly, 2006; Ragot et al., 2020), or enjoyment of the artwork (Moffat and Kelly, 2006). This is also the case of our experiments, as we assess the emotion experienced and the sensitivity attributed to the artist.

There are also differences among studies in the type of art evaluated. Hong et al. (2020) and Moffat and Kelly (2006) collected the participants’ judgments on AI music, while Chamberlain et al. (2018), Hong and Curran (2019), and Ragot et al. (2020) on AI painting. These investigations share some aspects of their procedure as well. They all show artworks created by AIs to one group of participants while the other group evaluates artworks created by human artists. We believe that this design does not allow researchers to know whether it is the prejudices about the authorship of the artwork that cause the differences in judgments or whether it is the artwork itself what is qualitatively different. It is for this reason that in our Experiment 1 both groups evaluated the artwork of an AI and we will use this procedure again in the present experiment. In addition, we will incorporate in this experiment some of the aforementioned measures, in order to facilitate comparisons between studies and to better consolidate the results obtained.

In this new experiment, we simplified the artistic stimuli. Instead of using the video artwork, which combined music and painting, we now used a purely musical artwork. In addition, we added a new, final, phase at the end of the experiment. Its purpose was to evaluate whether the participants would hold on to their judgments when we told them that, unlike what they were initially told, the author was actually human (or an AI, depending on the group; see details in the procedure section).

As in Experiment 1, we expected that emotionality and sensitivity would receive lower ratings when participants know that the artist was an AI than when they were told that the artist was a human.

### Method

#### Participants and Materials

We recruited 250 participants (47.6% women, 0.8% non-binary) over 18 years of age (*M* = 26.6, SD = 8.62) through the Prolific Academic platform. The sensitivity analysis for this sample size, very similar to that of the previous experiment, showed that we had a power of 80% to detect small effects (*d* = 0.31). The participants were randomly assigned to one of two groups: IA artist (*n* = 125), and human artist (*n* = 125). As in the previous experiment, the artwork shown to all participants was identical, but this time it was a purely musical piece performed by an IA.^3^ This experiment was conducted in English and was preregistered in AsPredicted: https://aspredicted.org/fh2u2.pdf.

#### Design and Procedure

After accepting the online informed consent, participants read different instructions for each group. Group AI artist was told that they would listen to a piece of music composed and performed by an artificial intelligence, while human artist group was simply told that the piece was composed and performed by a (human) artist (without specifying the gender and the human condition of the artist). We did not explicitly mention the term “human” because it might raise suspicions about the purpose of the study in the participants, as suggested by Hong and Curran (2019), and because if people are not warned about the potential AI authorship, they assume by default that the artist is human, as can be seen in Chamberlain et al. (2018).

In order to prevent participants from continuing through the questionnaire by mistake if the audio file was not loaded immediately, and also to ensure that at least part of the piece of music was listened to, the button to move on to the next page did not appear until 35 s had elapsed.

After listening to the artwork, all participants were asked to rate the emotion evoked by the music and the sensitivity they attributed to the artist, using the same questions as in Experiment 1. In addition, in this experiment we collected another extra measure on emotion, using the prototypical aesthetic emotions scale from Schindler et al. (2017). This is a 5-point scale that evaluates the intensity with which an emotion is felt and includes items, such as fascination, awe, or liking, used in Moffat and Kelly (2006) and Ragot et al. (2020). This allowed us to complement the one-question emotion measure that we used in Experiment 1 and in this experiment. We also asked participants to rate the quality of the artwork listened to (“*What was the quality level of the artwork?*”) on a scale labeled from 0 to 10.

Then, participants indicated their degree of agreement with Hong et al.’ (2020) questions on attitudes toward creative AI. That is, their discomfort with the presence of AI in art (*“Artificial intelligence that can perform artworks better than humans makes me uncomfortable”;* and *“I feel bad about myself if I consume art performed by artificial”*), and their judgment on how necessary it was to possess some human skills to compose music (*“Composing music is a task that requires the possession of human emotions”*; and *“Composing music is a task related to, and a very important part, of what it means to be human”*).

Next, we asked about participants’ gender and age, and showed them two binary questions, one about their experience with artificial intelligence and technology (“*Are your work or studies related to technology, artificial intelligence, robots or algorithms?*”) and one about their experience with music (“*Are your work or studies related to music?*”). In addition, they rated to what extent they liked classical music, because their preference for the musical genre of the experiment could condition their ratings according to Hong et al. (2020).

Finally, before debriefing the participants we added a new phase with respect to Experiment 1. In this new phase, we added a cover story, which was opposite to the one that each group had received at the start of the experiment. Group AI artist was now told that the authorship of the musical piece was actually a human. Group human artist was told that the author of the artwork was actually an AI. Then, we asked participants to rate again the emotion evoked by the artwork and the sensitivity attributed to the artist, using the same one-item questions they filled out before. The purpose of this phase was to evaluate whether changing the cover story would induce a difference in the subjective ratings of the experience.

### Results and Discussion

The results of this experiment replicated those of Experiment 1 with respect to the variable of artist’s sensitivity. The *T-Student*^4^ tests for independent samples confirmed that participants attributed stronger sensitivity to the artist when they believed that the artist was a human (*M* = 6.90, SD = 1.73) than in the AI artist group (*M* = 5.53, SD = 2.39; *t*(248) = 5.19, *p* < 0.001, *d* = 0.66). However, unlike in Experiment 1, participants did not indicate less emotion when they knew the artwork was performed by an AI (*M* = 5.18, SD = 2.43) than when they believed it was performed by a human artist [*M* = 5.58, SD = 2.02; *t*(248) = 1.39, *p* = 0.083, *d* = 0.18]. It is noteworthy that the mean emotion reported by participants in both groups has increased with respect to the previous experiment (*M_Human Artists_* = 4.09, *SD_Human Artists_* = 2.73, and *M_AI artist_* = 3.17, *SD_AI artist_* = 2.45 in Experiment 1). It is possible that this better reception of the current artwork could be affecting the differences between groups, although this might be due to many different factors.

Nevertheless, the differences on emotion become significant when we analyze the scores on the scale of prototypical aesthetic emotions. The scores on the scale, which had good internal consistency (*α* = 0.91), indicated a higher emotion in the human artist group (*M* = 3.09, SD = 0.85) than in the AI artist group [*M* = 2.90, SD = 0.90; *t*(248) = 1.72, *p* = 0.043, *d* = 0.22]. On this line, we also found differences between the two groups in the assessments of quality. Again, participants in the human artist group (*M* = 7.33, SD = 1.66) rated the quality of the artwork higher than those in the AI artist group [*M* = 6.33, SD = 1.99; *t*(248) = 4.31, *p* < 0.001, *d* = 0.55].

As we previously mentioned, according to Hong et al. (2020), the acceptance of creative abilities in AI would be a necessary requirement for its positive evaluation. We therefore analyzed whether this measure correlated with the variables reported previously: emotion, sensitivity, and quality of the artwork. We found a positive correlation between the acceptance of creative abilities in AI and emotion (as measured with the one-item question, *r* = 0.27, *p* < 0.001; and as measured with the scale of prototypical aesthetic emotions, *r* = 0.29, *p* < 0.001). We also observed a positive correlation between acceptance of creativity and sensitivity attributed to the artist (*r* = 0.17, *p* = 0.004); and between acceptance of creativity and the assessment of the quality of the artwork (*r* = 0.21, *p* < 0.001).

Furthermore, participants did not express high discomfort with the presence of AI in art (*M* = 3.45 out of 10, SD = 2.49; internal consistency of the two discomfort items, *α* = 0.70), although they did feel that composing music involves possessing human skills, such as emotion (*M* = 6.52 out of 10, SD = 2.43; consistency of the two skills items *α* = 0.75). Although these two variables did not correlate with our variables of emotion, sensitivity, or quality of the artwork (*ps* > 0.05), we found a negative correlation between acceptance of creativity in AIs and feeling discomfort with them (*r* = −0.34, *p* < 0.001), and between acceptance of creativity and understanding art as essentially human (*r* = −0.24, *p* < 0.001). In summary, the more AIs are seen as creative, the less discomfort people feel with AIs performing such work, and the weaker the strength with which art is associated with human skills.

Finally, we analyzed whether switching the information about the authorship of the artwork at the end of the experiment (telling the AI artist group that the artist was human, and the human artist group that the artist was an AI) affected their reported emotion and sensitivity scores. We performed 2 mixed ANOVAs with emotion and sensitivity scores as the dependent variables, the moment of measurement (i.e., before and after the original information was changed to opposite information about authorship) as the within-subjects factors, and group as a between-subjects factor. With respect to emotion we found no main effects of the moment of measurement [*F*(1, 248) = 0.78, *p* = 0.378, $\eta_{p}^{2}$ = 0.003], or group [*F*(1, 248) = 1.75, *p* = 0.187, $\eta_{p}^{2}$ = 0.007], nor a Moment of measurement x Group interaction [*F*(1, 248) = 0.09, *p* = 0.769, $\eta_{p}^{2}$ = 0.000]. That is, the emotion reported did not change after the participants were told that the author was different than they had initially been told (i.e., human or A.I.).

**Figure 2:**
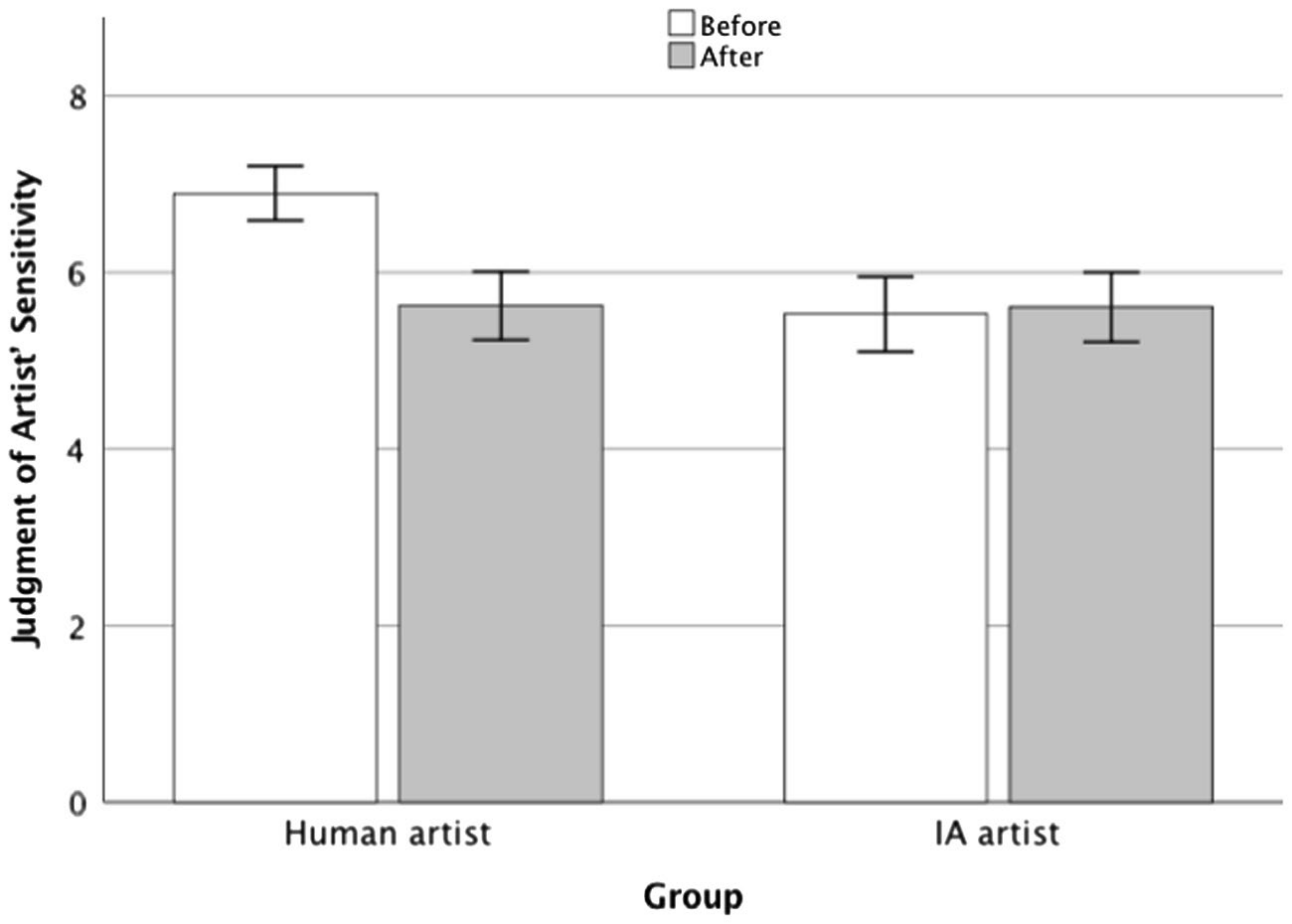
Judgment of artist’ sensitivity before and after receiving contradictory authorship information. Error bars represent 95% CI. The figure shows the sensitivity attributed to the artist in each group at baseline, as well as the sensitivity attributed after receiving contradictory information (AI authorship in the human group and human authorship in the AI group).

We did observe differences in sensitivity (see Figure 2). There was a main effect of moment of assessment [*F*(1, 248) = 29.3, *p* < 0.001, $\eta_{p}^{2}$ = 0.106], and group [*F*(1, 248) = 7.82, *p* = 0.006, $\eta_{p}^{2}$ = 0.031], as well as a Moment of assessment x Group interaction [*F*(1, 248) = 37.7, *p* < 0.001, $\eta_{p}^{2}$ = 0.132]. Pairwise comparisons showed that participants in the human artist group attributed significantly more sensitivity to the artist before the opposite information was introduced. As can be seen in Figure 2, participants in this group attributed more sensitivity at first, when they believed the artist was human, than once the cover story was changed and they were told that the author was an AI [*t*(248) = 8.17, *p* < 0.001, *d* = 0.65]. There were no statistically significant differences in the AI artist group [*t*(248) = −0.51, *p* = 0.956, *d* = −0.03].

As there were not many music experts in the sample (*n* = 23), we were not able to analyze whether their expertise influenced their judgments of emotion and sensitivity. What we did find was that, as Hong et al. (2020) noted, liking for the musical genre that we used in the experiment correlated positively with emotion (as collected in the one-item question, *r* = 0.41, *p* < 0.001; and on the scale of prototypical aesthetic emotions, *r* = 0.31, *p* < 0.001). It also correlated with sensitivity attributed to the artist (*r* = 0.25, *p* < 0.001) and the reported rating of the quality of the artwork (*r* = 0.15, *p* = 0.008).

In sum, our results replicate and extend the results of Experiment 1. Participants attribute less sensitivity to the artist when they know it is an AI than when they believe it is a human. Although we did not replicate the effect found in Experiment 1 on emotion captured with the one-question measure, we did observe differences in emotion between groups when measured with a more sensitive scale, the 8-item scale of prototypical aesthetic emotions. The absence of emotion reported between groups in the one-question measure in this experiment may be related to our having used a different artistic piece (only musical in this experiment versus musical and pictorial in the previous one). In fact, the piece in this experiment obtained higher ratings than the piece in the previous experiment, which is not surprising because AI techniques in this field of musical composition have improved substantially in the time between experiments (1 year and 10 months).

The reactions of the participants to our modification of the cover story at the end of the experiment supports the idea that prior expectations about AI influence the assessment of its performance. Participants modified their attribution of sensitivity when they “discovered” that the artwork was actually created by a human. Furthermore, according to our data, participants who attributed creative abilities to AIs also showed less discomfort with the presence of AI in the arts and regarded human skills as less necessary in this area.

Our results also replicate the results found by Hong et al. (2020) that attribution of creative abilities to AI and liking for the genre of the music evaluated do affect the quality assessment of the artwork. In our experiment, we additionally extend these findings by showing that they also affect reported emotion and sensitivity attributed to the artist. And we do so with an experimental design where it is evident that the judgment of the participants is due to the information provided about the authorship of the artwork and not to the artwork itself.

## General Discussion

Our results offer empirical evidence that, as already noted by David Cope (Johnson, 1997), the value of an artwork does not rest on the piece itself (as in our experiment it was the same for both groups), but on the subjective perceptions and attributions of the audience and their prior beliefs about the artistic skills of the author. Knowing that artificial intelligence has been the author of a piece of music seems to reduce the emotion experienced with it, the assessment of the artist’s sensitivity, and the assessment of the quality of the work.

Our results showed something that can be actually described as a form of cognitive bias affecting the evaluation of music. Cognitive biases are irrational modes of thought that occur in most people under similar circumstances and that affect decisions and preferences in all areas of our life (Kahneman, 2011). In this case, our results show a negative bias in the assessment of art created by AIs, and a default preference toward human artists. Perhaps knowing about the AI authorship could have activate certain negative traits or stereotypes about AI in the participants (Sundar, 2020), driving them to consider that AI does not have the necessary capabilities to emote and convey sensitivity with its art. In short, that AI cannot perform the subjective task of composing music. Similarly, and as we previously noted, in the area of decision-making Agudo and Matute (2021) also observed that people did not accept the explicit recommendation of algorithms in a subjective and emotional decision, such as who to date, but they did accept algorithmic explicit recommendations on apparently rational decision, such as who to vote for in a (fictitious) election.

In any case, it would be interesting for future research to explore additional venues for this potential undervaluation. For instance, Castelo et al. (2019) suggest that the rejection of artificial intelligence might also be triggered by the scarce presence of algorithms in some environments, by previous beliefs and prejudices about the skills of AI, and by the restlessness caused by the idea that AI can perform tasks which up to now had only been performed by humans. With respect to that latest point, two categories of human skills that can be projected (or not) on machines have already been proposed (Haslam, 2006; Loughnan and Haslam, 2007). First, attributes of human uniqueness, which distinguish humans from other animals but which are accepted to be shared with machines (i.e., usually of a cognitive nature, such as rationality and logic). Second, skills of human nature, which are assumed to be shared with other animals but not with machines (i.e., those that are emotional in nature, as well as intuition and imagination).

In the field of music, certain attributes, such as creativity, sensitivity, emotion, and even “soul” (Adams, 2010), which are usually assumed to be exclusive to humans and absent from machines, are considered essential requirements for quality execution. Therefore, the fact that machines show these abilities could lead to a worse assessment of the musical compositions generated by AI as observed in our experiments.

The present study contributes to our understanding of the sensitivity and the type of creative and emotional abilities people attribute to AI when valuing its artwork. As Hong et al. (2020) pointed out in their work, prior attitudes toward creative AI could be affecting appraisals of the artwork and the artist. Our experiments have replicated this with an experimental design where it is clearly demonstrated that the effect found is due to preconceptions about the artist’s skills and not the artwork, given that the artwork shown was the same for all participants. Probably, art is not a field in which the expected objectivity and neutrality of AI may add any positive or extra value to the art piece over what humans already contribute. However, we feel that there is often a tendency to turn the issue of AI’s creative abilities into a dichotomous question: either AI is creative or it is not. We believe, however, that creative capacity, both in humans and machines, should be treated as a gradient. Moreover, the growing presence of AI in art, generating creative pieces, often in collaboration with human artists, is proof that we are not facing a question with a dichotomous answer. Soon, this creative capacity of AI will probably not even be questioned, or, if it were, it would quite possibly be discussed in terms of a gradient.

Even so, it appears that there is still a way to go until the active presence of artificial intelligence in so exclusively human domains as art and music becomes familiar and valued. Perhaps the inflection point might come when “authentic humanity” (Adams, 2010) ceases to be considered an indispensable requirement for artistic creation.

### Pre-registration

Experiment 2 was pre-registered at AsPredicted.org: https://aspredicted.org/fh2u2.pdf.

## Data Availability Statement

The data and materials for this study can be found at Open Science Framework at: https://osf.io/3r7xg/.

## Ethics Statement

The Ethics Review Board of the University of Deusto approved the procedure for these experiments as part of a larger project on The Influence of Algorithms on Human Decisions and Judgements. Written informed consent was not requested because the research was online and harmless, participation was anonymous, and participants submitted their responses to the questionnaires voluntarily. No personal information was collected.

## Author Contributions

UA, MA, KL, and HM conceived and designed the experiments and revised the manuscript. UA contributed to experimental software. KL contributed to the artwork & AI. UA and MA conducted the experiments. UA and HM analyzed the data and wrote the manuscript. All authors contributed to the article and approved the submitted version.

## Funding

Support for this research was provided by Grant PSI2016-78818-R from Agencia Estatal de Investigación of the Spanish Government, as well as Grant IT955-16 from the Basque Government. The funders had no role in study design, data collection and analysis, decision to publish, or preparation of the manuscript.

## Conflict of Interest

The authors declare that the research was conducted in the absence of any commercial or financial relationships that could be construed as a potential conflict of interest.

## Publisher’s Note

All claims expressed in this article are solely those of the authors and do not necessarily represent those of their affiliated organizations, or those of the publisher, the editors and the reviewers. Any product that may be evaluated in this article, or claim that may be made by its manufacturer, is not guaranteed or endorsed by the publisher.

## Footnotes

^1^The video shows the digital artistic installation with artificial intelligence, “Water Color Melody Machine,” by one of the authors (KL.): https://player.vimeo.com/video/325421701.

^2^We also indicate the results with Welch’s *T*-Test because the assumptions of normality were violated according to the corresponding tests. However, since the sample is sufficiently large, the differences are minimal. Emotion [*t*(246) = 2.79, *p* = 0.003, *d* = 0.35]. Sensitivity [*t*(240) = 5.37, *p* < 0.001, *d* = 0.68].

^3^The listened artwork is available at http://bit.ly/2Onl4DF. It is a shorter version of the original piece a1_98137.mid, generated by the AI Music Transformer (https://magenta.github.io/listen-to-transformer/#a1_98137.mid). The artworks used in the experiment are also available in the Open Science Framework project for this research, at: https://osf.io/3r7xg/.

^4^As in the Experiment 1, we also indicate the results with Welch’s *T*-Test because the assumptions of normality and heteroscedasticity were violated according to the corresponding tests. Again, the differences are minimal. Sensitivity [*t*(226) = 5.19, *p* < 0.001, *d* = 0.66]. Emotion [*t*(240) = 1.39, *p* = 0.083, *d* = 0.18].
